# Dependence of O_2_ Depletion on Transition Metal Catalyst in Radical Polymerization of Cross-Linking Alkene Resins

**DOI:** 10.1021/acs.inorgchem.5c00760

**Published:** 2025-04-05

**Authors:** Hugo den Besten, Yanrong Zhang, Linda E. Eijsink, Andy S. Sardjan, Anouk Volker, Wesley R. Browne

**Affiliations:** Molecular Inorganic Chemistry, Stratingh Institute for Chemistry, Faculty of Science and Engineering, University of Groningen, Nijenborgh 3, 9747 AG Groningen, The Netherlands

## Abstract

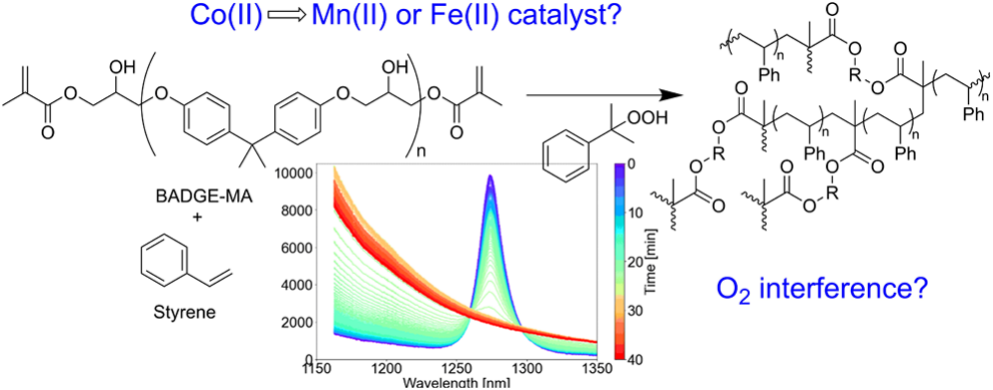

Cobalt(II) carboxylates show broad reactivity with peroxides and O_2_ and are the industry standard catalyst for the activation of peroxide initiators for the radical polymerization of alkenes under ambient conditions. Curing alkene-based resins containing cross-linking units, i.e., monomers containing two or more alkene units, is important in forming hard protective coatings and materials. The activation of peroxide initiators produces the propagating chain end radicals needed for polymerization. Since polymerization progress depends on the rate of initiator activation and the concentration of propagating radicals, interception of radicals by O_2_ can inhibit curing. Cobalt(II) carboxylates are used due to their reactivity in the presence of oxygen, even in resin coatings. Alternative catalysts based on manganese and iron are desirable. Hence, the impact of O_2_ on their performance in resin curing is of interest. Here, we use NIR emission and time-resolved spectroscopy, employing the O_2_-sensitive probe [Ru(ph_2_phen)_3_]^2+^, to determine the concentration of dissolved [O_2_] in alkene resins during curing with three representative catalysts, Co(II)(2-ethylhexanoate)_2_, Fe(II)-bispidine, and Mn(II)(neodecanoate)_2_. The rate of depletion of O_2_ is highly dependent on the catalyst used, but in all cases, it is well before the onset of the autoacceleration of polymerization in cross-linking resins.

## Abstract 2

In this study, we use NIR emission and time-resolved spectroscopy, employing the O_2_-sensitive probe [Ru(ph_2_phen)_3_]^2+^, to determine the concentration of dissolved O_2_ in alkene resins during curing with three representative catalysts, Co(II)(2-ethylhexanoate)_2_, Fe(II)-bispidine, and Mn(II)(neodecanoate)_2_. The rate at depletion of O_2_ is highly dependent on the catalyst used, but in all cases, it is well before the onset of the autoacceleration of polymerization in cross-linking resins.

## Introduction

The in situ formation of a solid material by polymerization of liquid precursors (e.g., a resin) is central to many applications in materials science, from 3D-printing and resin-molding to dental fillers and protective coatings, and typically uses initiated polymerization of monomers (alkenes, epoxides, etc.) together with cross-linking agents.^1^ Free radical polymerization of alkenes is controlled typically using initiators, which are frequently alkyl (hydro)peroxides.^2^ Radicals are generated by thermal decomposition (heating to 80–100 °C), at ambient temperatures by photolysis (UV light), or metal-catalyzed decomposition. The choice of the initiation method depends on the specific application, e.g., photolysis is used in dental applications.^3^ Cross-linked alkene-based resins are used widely as protective coatings and in molding. Initiation by metal-catalyzed decomposition of alkyl (hydro)peroxides is used under ambient conditions.^2,4,5^

**Figure 1:**
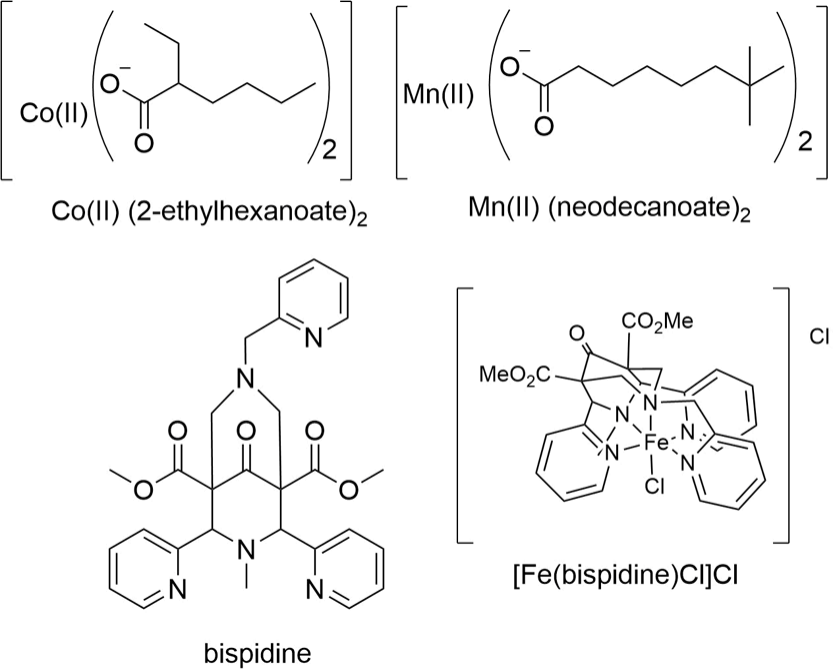
Structures of the catalysts, Co(II)(2-ethylhexanoate)_2_, Mn(II)(neodecanoate)_2_, and Fe(II)-bispidine, discussed in the text.

Cobalt(II) carboxylates are the current industry standard catalysts in room-temperature alkene-based resin curing, catalyzing the decomposition of cumene hydroperoxide to radical species, which then initiate polymerization. In recent years, efforts have been directed toward replacing cobalt(II) carboxylates with environmentally benign catalysts, specifically those based on iron(II) and manganese(II) (Figure 1).^6,7^ While these catalysts can activate alkyl hydroperoxide initiators, the impact of dissolved O_2_ is of substantial interest.^8^ Dissolved O_2_ is a known inhibitor in the free radical polymerization of alkenes.^9−14^ The inhibition is attributed to the formation of less reactive ROO· radicals from the reaction of R· with O_2_.^9,15,16^ Therefore, dissolved O_2_ in resins can impact polymerization (curing) kinetics due to both interference with radical propagation reactions^8^ and, in some cases, interaction with catalysts, as shown recently by Anastasaki and co-workers in the case of copper-catalyzed ATRP reactions.^17^ Purging to remove O_2_ avoids such interference but is unfeasible on a large scale and when resins are applied to form protective coatings under ambient conditions.

**Scheme 1:**
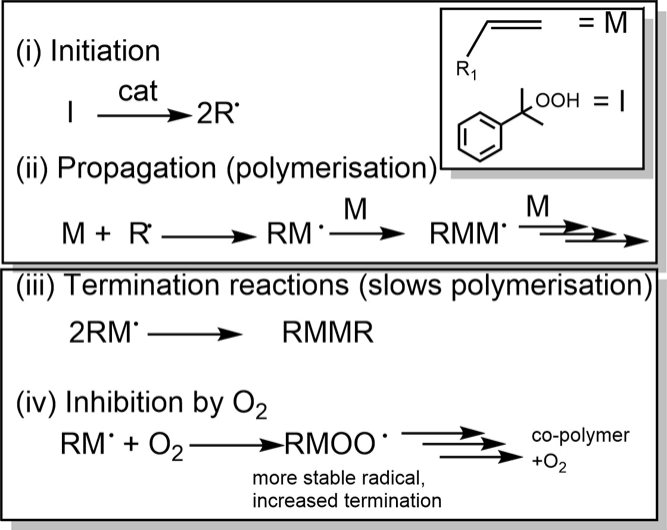
Transition Metal Catalysts Initiate Polymerization (i) by Decomposing Cumene Hydroperoxide into Radicals that (ii) Initiate the Polymerization of Alkenes The polymerization is interrupted by (iii) growing chains reacting with each other and (iv) with O_2_. For a detailed discussion of the relative rates, see the SI.

Additives, e.g., tertiary amines, are used in commercial formulations to scavenge O_2_, and the oxygen radicals formed and they have been optimized to work with cobalt(II) carboxylate catalysts over the last half century. This codevelopment presents challenges in efforts toward Co(II) replacement (e.g., interference or deactivation of Fe(II) and Mn(II) catalysts by additives) since both Co(II) and Co(III) salts can react with alkyl-oxy and -peroxy radicals in a manner that is unique among transition metals.^18,19^ Furthermore, curing alkene-based resins using alkyl hydroperoxides (typically cumene hydroperoxide) is characterized by a significant lag period before the onset of polymerization, i.e., autoacceleration (see Scheme 1 and the SI for further details).^20^ The lag phase needs to be long enough to allow for the application of coatings to surfaces but cannot be too long due to the loss of reactive diluents (e.g., styrene) over time by evaporation. These characteristics place considerable restrictions on the development of catalyst replacements. The relation between [O_2_] and the lag phase observed during alkene resin curing with cobalt(II) carboxylates and their prospective replacements is therefore of interest.

**Figure 2:**
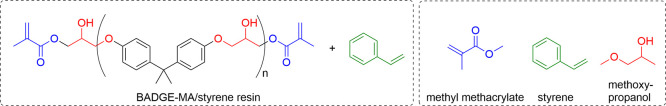
Left: BADGE-MA/styrene resin mixture; right: styrene/MMA/MeOPrOH mixture used as a model, consisting of styrene, methyl methacrylate, and 1-methoxy-2-propanol (1:1:1 vol. ratio).

In the present contribution, interference by O_2_ in the curing of a representative additive-free alkene resin, comprising styrene and the cross-linker BADGE-MA (Figure 2), with two potential Co(II) carboxylate replacements, Mn(II)(neodecanoate)_2_ and Fe(II)-bispidine (Figure 1), is investigated.^21^ The cross-linker BADGE-MA is a representative example of the bifunctional cross-linkers used in commercial alkene-based resin mixtures. It forms cross-linked polymers together with a reactive diluent such as styrene; Figure 2. The role of O_2_ in the delay to the onset of autoacceleration following the addition of an initiator to alkene-based resins is explored. Inline spectroscopy is used to determine the change in [O_2_] over time in both BADGE-MA/alkene resins and in model non-cross-linking alkene mixtures (Figure 2).

**Figure 3:**
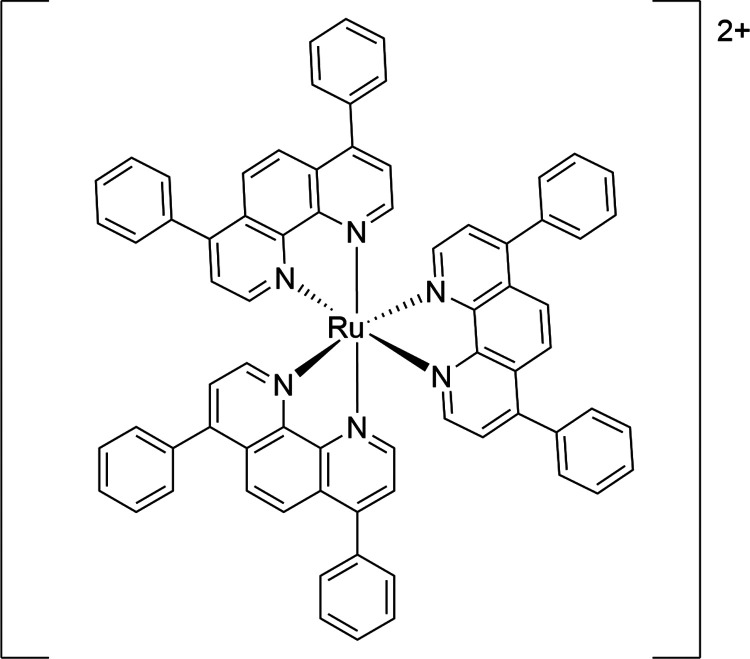
Structure of the luminescent probe [Ru(ph_2_phen)_3_]^2+^.

Changes in [O_2_] are determined through dynamic quenching of the emission of the complex [Ru(ph_2_phen)_3_]^2+^ (where ph_2_phen is 4,7-diphenyl-1,10-phenanthroline, Figure 3). Interference by emission quenchers, e.g., the catalysts and species formed during the curing, is determined using the sensitized NIR phosphorescence of ^1^O_2_^22^ together with the observed emission decay rate of the sensitizer [Ru(ph_2_phen)_3_]^2+^.^23^ Additionally, the exchange of O_2_ between the resin and air is determined by headspace Raman spectroscopy. Together, the data allows for the relation between the presence of O_2_ and the lag period before the onset of autoacceleration (rapid polymerization) to be established under a wide range of conditions, with the reference cobalt(II) catalyst and the potential replacement Mn(II) and Fe(II) catalysts (Figure 1).

## Results and Discussion

**Figure 4:**
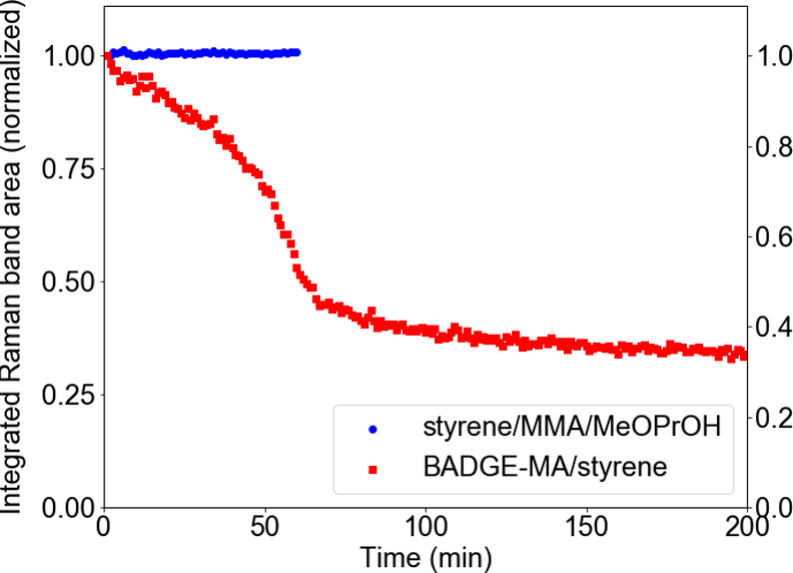
Integrated area of the ν_*C*=*C*,*str*_ (normalized to the initial area) Raman band at 1630–1637 cm^–1^ over time following addition of cumene hydroperoxide to a mixture of (Co(II)(2-ethylhexanoate)_2_) and (red) BADGE-MA/styrene (1.0 g/0.34 g), and (blue) styrene/MMA/MeOPrOH (styrene, methyl methacrylate, and 1-methoxy-2-propanol, 1:1:1 vol. ratio) at 19 °C. Significant alkene conversion in the model mixture is not observed within 24 h. Data are normalized to the area of the Raman band corresponding to the alkene C=C stretching vibration (1630–1637 cm^–1^).

The resin mixture studied here is composed of the reactive diluent styrene and the cross-linker BADGE-MA (Figure 2). The preparation and characterization of BADGE-MA have been reported earlier^24^ and are used here to ensure a known chemical composition and residual acid content. Although the mixture is a simplification of commercial resins, the curing profile (Figure 4) exhibits the expected lag period, i.e., slow consumption of alkene, followed by a period of autoacceleration (Trommsdorff effect)^20^ in which the rate of alkene polymerization accelerates until the glass point is reached and curing is halted. A model solvent system comprising styrene, methyl methacrylate, and 1-methoxy-2-propanol (styrene/MMA/MeOPrOH) was selected to match closely the chemical composition and solvent properties of BADGE-MA/styrene, with 1-methoxypropan-2-ol both to provide the alcohol functional groups present in BADGE-MA and to ensure solubility of the catalysts used (Figure 2).

Although comparable with regard to functionality, the viscosity of this model mixture is lower than that of BADGE-MA/styrene, which impacts diffusion-controlled processes, i.e., radical–radical combinations, bimolecular quenching, etc., vide infra. The alkene polymerization in the model mixture is not observed over the first few hours after addition of the initiator, and indeed, even after 24 h, the extent of conversion is negligible. Therefore, the viscosity of the mixture remains relatively constant over the period of interest to the present study, i.e., the first few hours after addition of the initiator (Figure 4), and hence, molecular diffusion coefficients can be assumed to be constant over time. The only variable of concern in the photophysical studies described below is the concentration of the interacting species, namely, [Ru(ph_2_phen)_3_]^2+^ and quenchers, such as O_2_.

### Dependence of O_2_ Uptake/Release on the Catalytic Decomposition of Cumene Hydroperoxide

**Figure 5:**
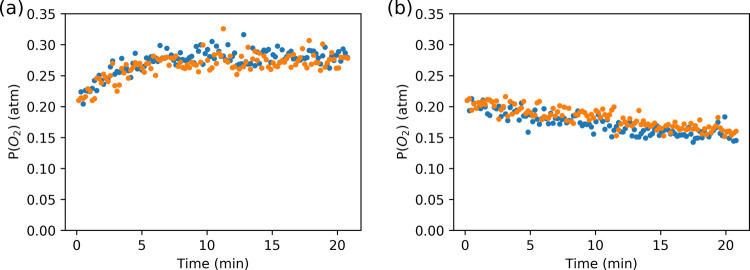
Time dependence of *P*(O_2_) in the headspace above (a) cyclohexane and (b) above styrene/methyl methacrylate (1:1 vol/vol) during the decomposition of cumene hydroperoxide by Co(II)(2-ethylhexanoate)_2_ monitored by Raman spectroscopy (λ_exc_ 785 nm). Orange/Blue indicate independent experiments.

The transition metal-catalyzed decomposition of alkyl peroxides, such as cumene hydroperoxide, to release O_2_ was studied earlier by Spier et al. in cyclohexane.^5^ The rapid equilibration of gases between the solution and the headspace above it allows for the release of O_2_ to be monitored using headspace Raman spectroscopy.^25,26^ The release of O_2_ into the headspace, following addition of cumene hydroperoxide to Co(II)(2-ethylhexanoate)_2_ in cyclohexane, proceeded as expected,^5^ corresponding to ca. 10 μmol of O_2_ (i.e., 10% with respect to the cumene hydroperoxide added) over 5 min (Figure 5).

**Figure 6:**
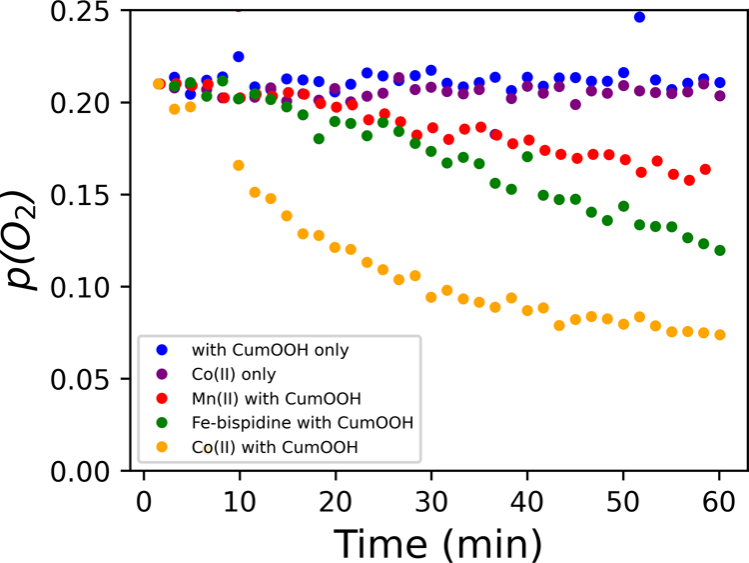
Time dependence of *p*_O_2__ in the headspace of a sealed vial after addition of cumene hydroperoxide (CumOOH) to styrene/MMA/MeOPrOH without a catalyst, and with Co(II)(2-ethylhexanoate)_2_, Fe(II)-bispidine, or Mn(II)(neodecanoate)_2_ and with Co(II)(2-ethylhexanoate)_2_ only. CumOOH, cumene hydroperoxide.

The decomposition of cumene hydroperoxide by Co(II)(2-ethylhexanoate)_2_ in a mixture of styrene and methyl methacrylate is relatively fast (determined iodometrically earlier^24^), and hence, the release of O_2_ into the headspace would be expected. However, the opposite is observed: O_2_ is removed from the headspace over time, indicating that O_2_ is consumed in solution (Figure 5). Indeed, in contrast to in cyclohexane, in styrene/methyl methacrylate, ca. 8 μmol of O_2_ was consumed from the headspace at a steady rate over 20 min (Figure 5), corresponding to the period of rapid decrease in the concentration of cumene hydroperoxide (Figure S1). Together with the estimated initial [O_2_] in the mixture of 3.9 mM (see Table S1), this change corresponds to a stoichiometry of ca. 0.1:1 with respect to the cumene hydroperoxide added. Surprisingly, although neither Fe(II)-bispidine nor Mn(II)(neodecanoate)_2_ show substantial decomposition of cumene hydroperoxide over the first hour (Figure S1),^6^ the observed rate and extent of consumption of O_2_ are only marginally lower than with Co(II)(2-ethylhexanoate)_2_ (Figure 6). Therefore, the reaction with O_2_ is not directly correlated with the extent and rate at which the catalysts decompose cumene hydroperoxide.

Hence, depletion of dissolved O_2_ in the resins would be expected in the first minutes also. However, although equilibration of O_2_ between solution and headspace is relatively rapid for cyclohexane and mixtures of styrene and methyl methacrylate, such equilibration is slow in the more viscous BADGE-MA/styrene resin, with only the first millimeters of solution in contact with the headspace showing significant exchange (vide infra). Hence, the in situ determination of dissolved [O_2_] is required.

### In Situ Determination of O_2_ Using [Ru(II)(ph_2_phen)_3_](PF_6_)_2_

In situ determination of [O_2_] in alkene mixtures, which do not contain cross-linking monomers, is possible, e.g., using a Clark probe or optical oxygen probe sensors.^27^ In resins containing cross-linkers that undergo a large increase in viscosity and form hard solids, noncontact optical detection using a dispersed O_2_-sensitive luminescent compound is advantageous.^28−30^ The quenching of emission by O_2_ is dynamic and hence dependent on diffusion rate constants, as well as concentrations, and several studies have focused on accounting for these variables.^31−33^

The change in [O_2_] in the resin and model mixtures following addition of cumene hydroperoxide was determined indirectly through the emission decay lifetime (τ_obs_) of [Ru(II)(ph_2_phen)_3_](PF_6_)_2_.^34,35^ Quenching of the phosphorescence of [Ru(II)(ph_2_phen)_3_](PF_6_)_2_ by dissolved O_2_ results in a reduction in the former’s emission decay lifetime and a corresponding NIR emission from the ^1^O_2_ generated in the process (vide infra). The [O_2_] is directly related to the observed rate of decay (*k*_obs_) of the emission (dynamic quenching) through the Stern–Volmer equation, eq 1.
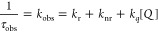
1where τ_obs_ is the observed emission lifetime, *k*_obs_ is the observed rate of emission decay, *k*_r_ is the radiative decay rate, *k*_q_ is the rate of quenching, and *k*_nr_ is the rate of other nonradiative decay paths.

However, as it is a diffusion-controlled process, it is also dependent on solvent viscosity (, where η is viscosity and *T* is temperature, in the Einstein–Stokes model), which differs considerably between the solvent mixture styrene/MMA/MeOPrOH and the BADGE-MA/styrene resin, with the latter much more viscous. Furthermore, the high viscosity of cross-linking alkene resins, even before curing has been initiated, poses an additional challenge due to the potential irreversible decrease in the concentration of O_2_ in the confocal volume of the spectrometer during the measurement. Hence, initial studies have focused on the chemically equivalent styrene/MMA/MeOPrOH mixture.

The [O_2_] in the model mixture is estimated at ca. 3.6 mM (Table S1),^25,36^ which is consistent with the emission lifetime of [Ru(II)(ph_2_phen)_3_](PF_6_)_2_ (315 ns) in the mixture. The emission lifetime increases to 4.8 μs after purging with N_2_ or argon, similar to that in other solvents at room temperature in the absence of O_2_.^37^ The addition of cumene hydroperoxide or any of the catalysts, Fe(II)-bispidine, Co(II)(2-ethylhexanoate)_2_, or Mn(II)(neodecanoate)_2_, does not affect the emission lifetime in an air-equilibrated solvent (Figure S2) even 1 h after addition. In contrast, a substantial increase in emission lifetime (τ_obs_) is observed after the addition of cumene hydroperoxide to mixtures of styrene/MMA/MeOPrOH containing any of the three catalysts, indicating that dissolved O_2_ is consumed, vide supra.

With Co(II)(2-ethylhexanoate)_2_ (Figure S2a), τ_obs_ increases to 4 μs within 1 min of addition of the cumene hydroperoxide due to a decrease in [O_2_] by at least 90% to <0.4 mM. After 1 h, however, τ_obs_ decreases again to ca. 2.5 μs. The subsequent decrease in τ_obs_ is not due to re-equilibration of O_2_ between the solution and the headspace (vide supra) since the same changes in τ_obs_ were observed in completely filled and sealed sample vials (i.e., with no headspace).

With Fe(II)-bispidine, τ_obs_ increases less rapidly (over 10 min) to almost 5 μs (Figure S2b). In the first minute, the emission decay is multiexponential due to the lifetime increasing during the time taken to acquire the data (ca. 2 min). At 5 min and 1 h, the emission decay is monoexponential. With Mn(II)(neodecanoate)_2_, τ_*obs*_ increases much more slowly, reaching the maximum emission lifetime only after 20 min (Figure S2c).

**Figure 7:**
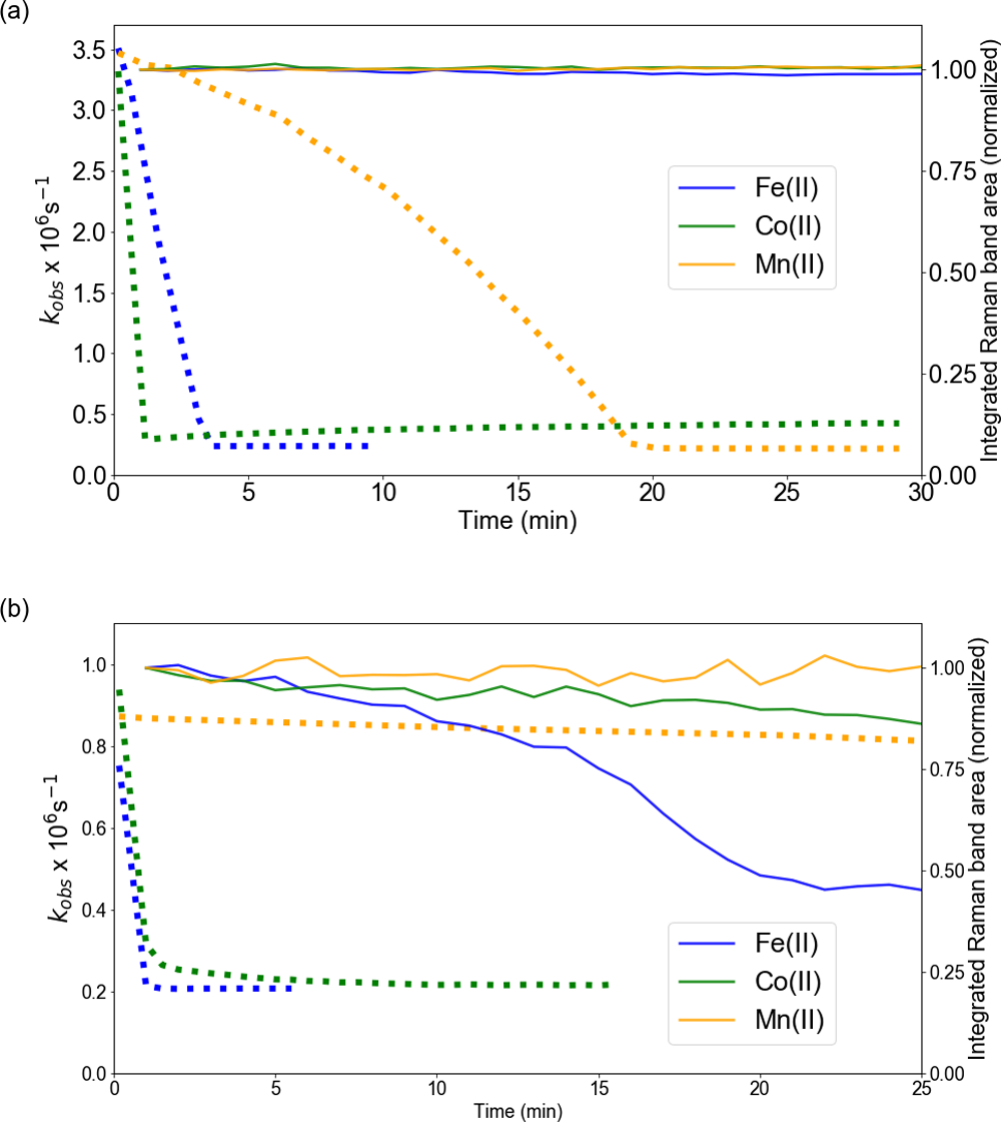
*k*_obs_ (squares) and integrated area of the ν_*C*=*C*,*str*_ (normalized to the initial area) Raman band at 1630–1637 cm^–1^ (line) over time after addition of cumene hydroperoxide (92 mM) with Fe(II)bispidine, Co(II)(2-ethylhexanoate)_2_, or Mn(II)(neodecanoate)_2_ (a) in styrene/MMA/MeOPrOH and (b) in BADGE-MA/styrene resin. See Figure S4 for a change over 200 min.

The change in τ_obs_ over time for the three catalysts is shown in Figure S3. Since the decay rate (*k*_obs_, eq 1), and not the emission lifetime, is linearly proportional to [O_2_], Figure 7 provides for a clearer overview of the change in [O_2_] over time.

The data together show that with Co(II)(2-ethylhexanoate)_2_ and Fe(II)-bispidine, the solution is depleted of O_2_ within a few minutes of addition of cumene hydroperoxide. The increase in the emission lifetime with Mn(II)(neodecanoate)_2_ is more gradual. Indeed, even after 5 min, the [O_2_] is essentially unchanged, and it takes up to 1 h after addition for the maximum lifetime to be reached.

The higher viscosity of the resin, compared to the model mixture, reduces the extent of quenching by O_2_, and hence, initially, the emission lifetime (τ_obs_) is longer, i.e., ca. 1.1 μs in the resin. Nevertheless, essentially the same changes in τ_obs_, and hence [O_2_], over time were observed with each of the catalysts and cumene hydroperoxide in the BADGE-MA/styrene resin. The increase in τ_obs_ occurred more rapidly in the resin with Fe(II)-bispidine and Co(II)(2-ethylhexanoate)_2_ than in the model mixture, and in both cases, the final emission lifetimes reached were longer. With Mn(II)(neodecanoate)_2_, a gradual increase in τ_obs_ was observed after the addition of cumene hydroperoxide with a rapid increase in τ_obs_ after ca. 100 min after.

The changes in emission decay rates (*k*_obs_) can be related directly with [O_2_]. It is apparent therefore (Figure 7) that with Co(II)(2-ethylhexanoate)_2_, Fe(II)-bispidine, and eventually Mn(II)(neodecanoate)_2_ in BADGE-MA/styrene, all O_2_ is consumed following addition of cumene hydroperoxide, in both cases, well before the onset of autoacceleration (manifested in a rapid decrease in the intensity of the Raman band (ν_*C*=*C*_) at ca. 1630 cm^–1^, Figure 7).

The decrease in τ_obs_ observed with Co(II)(2-ethylhexanoate)_2_ in the model mixture is likely not observed in more viscous resin since diffusion, and hence bimolecular quenching, has much less impact (vide infra). With Co(II)(2-ethylhexanoate)_2_ in styrene/MMA/MeOPrOH, the decrease in emission lifetime several minutes after it had reached a maximum indicates that a quencher forms in situ in the mixture over time. Given that O_2_ is liberated by the reaction of Co(II)(2-ethylhexanoate)_2_ with cumene hydroperoxide in cyclohexane (vide infra), it is possible that a steady state is reached where oxygen consumption and production balance. The lifetime reached (2.5 μs) would require the [O_2_] to reach 0.55 mM. However, the formation of another quencher should be considered too, i.e., Co(III) species (vide infra).

### Singlet Oxygen Emission

Quenching of the excited state of ruthenium(II) polypyridyl complexes by energy transfer to (^3^O_2_) results in the generation of singlet oxygen (^1^O_2_), which relaxes to the ground state (^3^O_2_) primarily by radiationless deactivation, but with some radiative decay resulting in phosphorescence at 1268 nm. Hence, the observation of changes in the intensity of the characteristic phosphorescence of ^1^O_2_ in the NIR region can confirm that the increase in the emission lifetime of [Ru(ph_2_phen)_3_]^2+^ is due to a decrease in [^3^O_2_]. These data supplement the emission lifetime data discussed above, and the data sets should be consistent if O_2_ is the only quencher involved.

**Figure 8:**
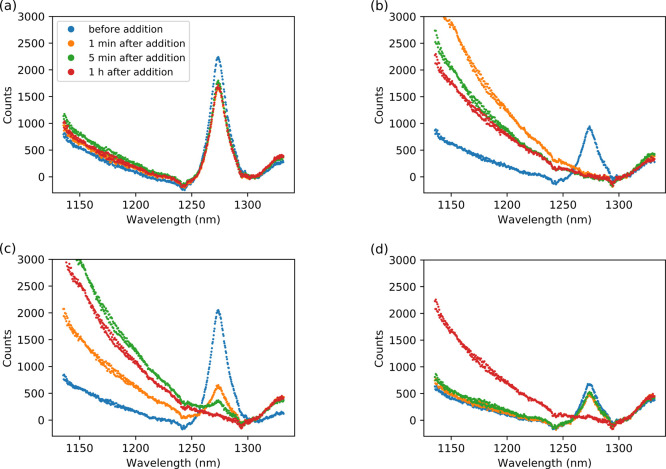
NIR emission spectrum of [Ru(ph_2_phen)_3_](PF_6_)_2_ in styrene/MMA/MeOPrOH (a) without a catalyst, and with (b) Co(II)(2-ethylhexanoate)_2_ (2 mM), (c) Fe(II)-bispidine (0.2 mM), or (d) Mn(II)(neodecanoate)_2_ (2 mM), before and at selected times after addition of cumene hydroperoxide (92 mM), (λ_exc_ 355 nm).

The NIR emission spectrum obtained upon excitation of [Ru(ph_2_phen)_3_]^2+^ in styrene/MMA/MeOPrOH shows the expected narrow emission band of ^1^O_2_ at 1268 nm, on top of the broad tail of the emission from [Ru(ph_2_phen)_3_]^2+^, allowing for changes in emission intensity for both species to be monitored simultaneously. Addition of cumene hydroperoxide resulted in a minor decrease in the intensity of ^1^O_2_ emission and a small increase in emission from [Ru(ph_2_phen)_3_]^2+^ (Figure 8a). The emission spectrum remained essentially unchanged for over 1 h thereafter, consistent with the absence of change in the emission lifetime of [Ru(ph_2_phen)_3_]^2+^ (Figure S2d).

Addition of either Co(II)(2-ethylhexanoate)_2_ or Mn(II)(neodecanoate)_2_ to styrene/MMA/MeOPrOH with [Ru(ph_2_phen)_3_]^2+^ resulted in a decrease in intensity of the emission of ^1^O_2_. The decrease in the case of Co(II)(2-ethylhexanoate)_2_ and Mn(II)(neodecanoate)_2_ is due to primary inner filter effects, i.e., absorbance by the solutions at the excitation wavelength used (355 nm). In contrast, addition of Fe(II)-bispidine, which does not absorb significantly at 355 nm, does not affect the emission spectrum. UV/vis absorption spectroscopy shows a further increase in absorption at 355 nm over time following addition of cumene hydroperoxide for solutions containing any of the three catalysts (Figures S5–S7).^38^

With Co(II)(2-ethylhexanoate)_2_ present, a complete loss in emission due to ^1^O_2_ was observed within 1 min of the addition of cumene hydroperoxide (Figure 8b). The tailing emission from [Ru(ph_2_phen)_3_]^2+^ increased and then decreased partly again, consistent with the decrease in the emission lifetime of [Ru(ph_2_phen)_3_]^2+^ between 5 min and 1 h after addition of cumene hydroperoxide (Figure S2a,b). Notably, the absence of emission at 1268 nm from ^1^O_2_ between 5 min and 1 h confirms that oxygen is not responsible for quenching of the emission of [Ru(ph_2_phen)_3_]^2+^ and therefore is no longer present (<10%; see the Experimental Section for details). Furthermore, purging with argon (to remove any remaining O_2_) 1 h after the addition of cumene hydroperoxide has no effect on the emission lifetime.

With Fe(II)-bispidine, the decrease in emission intensity was slower (Figure 8c), with weak emission persisting for up to 5 min, consistent with the changes seen in the emission lifetime (Figure S2b).

**Figure 9:**
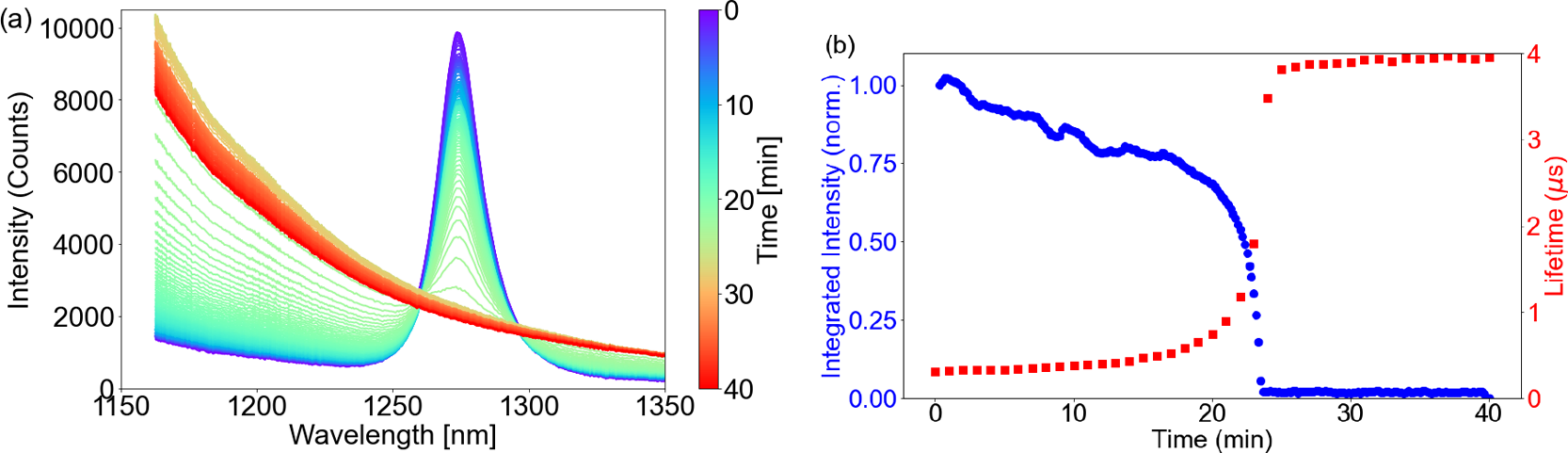
(a) NIR emission spectrum with [Ru(ph_2_phen)_3_](PF_6_)_2_ in styrene/MMA/MeOPrOH containing Mn(II)(neodecanoate)_2_ (2 mM), before (blue) and after addition of cumene hydroperoxide (λ_exc_ 450 nm), and (b) integrated area of emission at 1268 nm (of ^1^O_2_, blue) and τ_obs_ (of [Ru(ph_2_phen)_3_](PF_6_)_2_, red) over time.

With Mn(II)(neodecanoate)_2_, τ_obs_ increases slowly over time after addition of cumene hydroperoxide, corresponding well to the decrease in ^1^O_2_ emission observed (Figures 9 and S3). After 15 min, the rate of change in τ_obs_ and ^1^O_2_ emission increases rapidly, with eventually a complete loss in ^1^O_2_ emission.

#### Quenching of Emission with Cumene Hydroperoxide and Co(II)(2-Ethylhexanoate)_2_

The emission lifetime of [Ru(ph_2_phen)_3_](PF_6_)_2_ in the presence of Co(II)(2-ethylhexanoate)_2_ and cumene hydroperoxide changes in a more complex manner than with Fe(II)-bispidine or Mn(II)(neodecanoate)_2_. Specifically, the emission lifetime increases from 300 ns to 4.5 μs within 20–30 s of the addition of cumene hydroperoxide (vide supra, Figure S3), after which the emission lifetime decreases to 2.5 μs over 15–20 min. The latter decrease in emission lifetime indicates an increase in the concentration of a quencher in the mixture over time. The absence of emission from ^1^O_2_ (Figure S9) confirms that the [O_2_] does not increase again (vide supra). Furthermore, the emission decay lifetime of [Ru(ph_2_phen)_3_](PF_6_)_2_ was not affected by the presence of Co(II) in the model solvent mixture. Hence, the formation of Co(III) species in the reaction mixture over time was considered the most likely cause since quenching of the excited states of ruthenium(II) polypyridyl complexes by Co(III) salts (via oxidative electron transfer) is well-known.^39,40^

**Figure 10:**
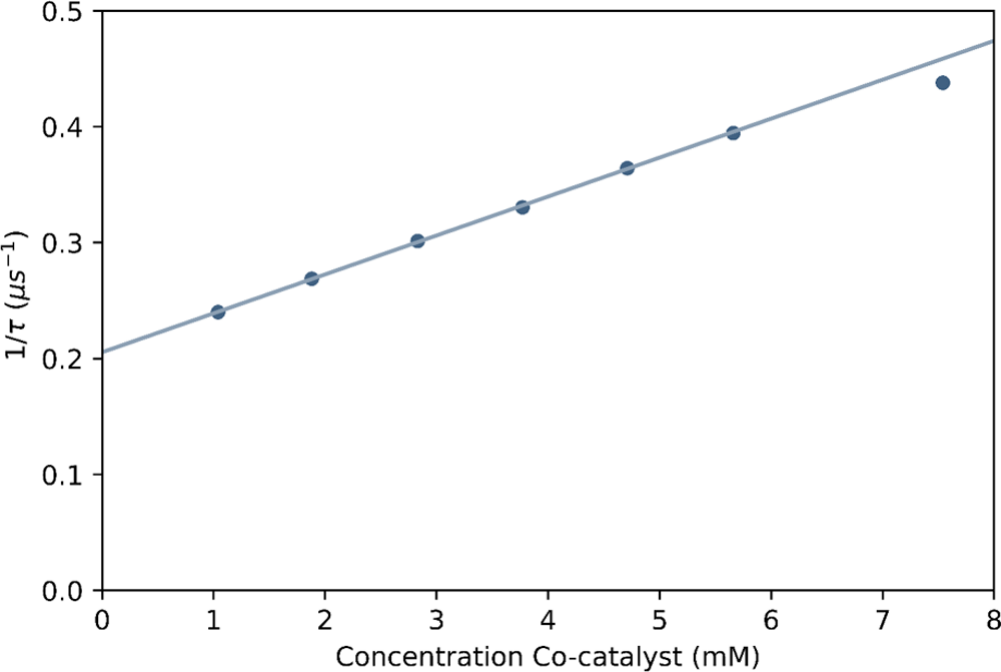
Dependence of 1/τ_obs_ for [Ru(ph_2_phen)_3_](PF_6_)_2_ in tertbutylstyrene/MMA/MeOPrOH containing increasing initial concentrations of Co(II)(2-ethylhexanoate)_2_. τ_obs_ was determined 15 min after the addition of cumene hydroperoxide (92 mM). Note that the lifetime is unaffected by the presence of Co(II)(2-ethylhexanoate)_2_ alone over this range of concentrations (Figure S10).

Indeed, a linear dependence of *k*_obs_, 15 min after addition of cumene hydroperoxide, on the initial concentration of Co(II)(2-ethylhexanoate)_2_ was observed (Figures 10 and S10),^41^ consistent with the oxidative electron transfer quenching of [Ru(ph_2_phen)_3_]^2+^ by Co(III) formed in situ. The quenching is consistent with changes in the UV/vis absorption spectrum of the reaction mixture (Figure S5), which indicates conversion from the Co(II) to Co(III) oxidation state also.^42,43^

#### NIR Emission Spectroscopy in BADGE-MA/Styrene Resins

The higher viscosity of the cross-linker containing resin (BADGE-MA/styrene) reduces the diffusivity of O_2_ and [Ru(ph_2_phen)_3_](PF_6_)_2_ and hence the intensity of the sensitized ^1^O_2_ emission. Nevertheless, the NIR emission of ^1^O_2_ was observed in the resin mixture. However, a rapid decrease in emission intensity at 1268 nm (^1^O_2_) and a corresponding increase in the emission intensity of [Ru(ph_2_phen)_3_](PF_6_)_2_ (within 2–3 s) are observed with laser excitation due to rapid depletion of the O_2_ in the confocal volume of the spectrometer (Figure S11).^44^ The high viscosity of the resin prevents rapid replenishment of O_2_ in the confocal volume. Continuous movement of the sample during acquisition of NIR emission spectra is nevertheless sufficient to allow for spectra to be recorded with minimum impact on intensity from the photoinduced consumption of O_2_.

**Figure 11:**
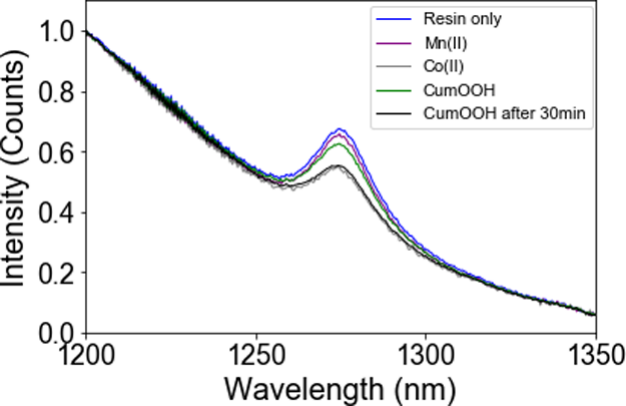
NIR emission spectra (λ_*exc*_ 450 nm) with [Ru(ph_2_phen)_3_](PF_6_)_2_ in BADGE-MA/styrene alone (blue) and with Mn(II)(neodecanoate)_2_ (purple), Co(II)(2-ethylhexanoate)_2_ (gray), and cumene hydroperoxide (initially (green) and after 30 min (black)). The spectra are offset-corrected at 1370 nm and normalized at 1200 nm. CumOOH, cumene hydroperoxide.

The NIR emission of ^1^O_2_ in the resin is unaffected by addition of Mn(II)(neodecanoate)_2_ and shows a modest initial decrease with the addition of Co(II)(2-ethylhexanoate)_2_, with no further change in either case over 2 h (Figure 11). Notably, the addition of cumene hydroperoxide to BADGE-MA/styrene reduces the emission intensity slowly over time, which indicates that O_2_ is depleted slowly even in the absence of a catalyst. Raman spectra recorded 2 h after addition of any of the components alone show that alkene polymerization has not occurred to a significant extent (Figure S12).

As observed in the model mixture (vide supra), the emission from ^1^O_2_ decreases within minutes of addition of cumene hydroperoxide to BADGE-MA/styrene with Co(II)(2-ethylhexanoate)_2_, and the expected^6^ eventual extent of alkene polymerization (at 19 °C) was observed (Figure S12).

**Figure 12:**
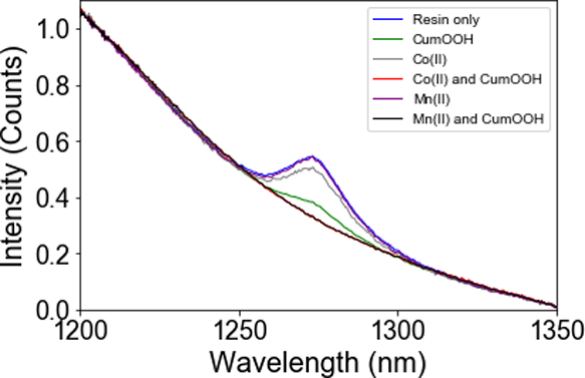
NIR emission spectra (λ_exc_ 450 nm) with [Ru(ph_2_phen)_3_](PF_6_)_2_ in BADGE-MA/styrene alone (blue) and with cumene hydroperoxide (green), with Co(II)(2-ethylhexanoate)_2_ (gray), with Co(II)(2-ethylhexanoate)_2_ and cumene hydroperoxide (red), with Mn(II)(neodecanoate)_2_ (purple), and with Mn(II)(neodecanoate)_2_ and cumene hydroperoxide (black). The spectra are offset-corrected at 1370 nm and normalized at 1200 nm and were recorded 24 h after the addition of cumene hydroperoxide. CumOOH, cumene hydroperoxide.

With Mn(II)(neodecanoate)_2_ and cumene hydroperoxide, the NIR emission decreased slowly over 2 h, similar to that observed with cumene hydroperoxide alone (Figure 12). Alkene polymerization was not observed over this time period although the extent of polymerization after 24 h was the same as that with Co(II)(2-ethylhexanoate)_2_ (Figure S12). Notably, the emission intensity did not decrease near the top of the sample (i.e., the resin in contact with the headspace), consistent with replenishment of O_2_ by diffusion from the headspace.

The data indicate that although some differences are observed between the model mixture styrene/MMA/MeOPrOH and the resin BADGE-MA/styrene, the reactivity with respect to the depletion of the O_2_ observed is equivalent in both mixtures.

## Conclusions

The replacement of Co(II) carboxylates with Fe(II) and Mn(II) catalysts is desirable, and in the present study, the catalysts were selected due to their use already as driers in alkyd-based paints.^50^ Of particular interest are the differences in the reactivity of O_2_ in the presence of these catalysts compared to that with Co(II)(2-ethylhexanoate)_2_. Knowledge of the rate of depletion of O_2_ during alkene-based resin curing is important due to the role that O_2_ can play in inhibiting polymerization. In the present study, we show that the luminescent probe [Ru(ph_2_phen)_3_]^2+^ can be used to track the depletion of dissolved O_2_ in real time during resin curing and that interferences by other quenchers (e.g., Co(III) species) can be accounted for by the simultaneous determination of emission from the ^1^O_2_ generated during the measurement, as a positive control. The data also show that chemically equivalent low-viscosity models for alkene resins can serve as accurate surrogates for more viscous cross-linking alkene resins, provided that the impact of differences in viscosity on bimolecular processes is taken into account.

O_2_ is consumed from the headspace of alkene mixtures through the reaction of cumene hydroperoxide with all three catalysts to a greater or lesser extent, with the most rapid uptake observed with Co(II)(2-ethylhexanoate)_2_. The high viscosity of the BADGE-MA/styrene resin limits the exchange of O_2_ with the headspace to the first microns of the resin surface, and hence, more rapid depletion and eventually a complete loss of dissolved O_2_ can be expected.

Importantly, with regard to the replacement of Co(II)-based catalysts, the depletion of dissolved O_2_ is shown to be dependent on the catalyst replacement used. However, in viscous cross-linking resins, depletion of O_2_ occurs in all cases well before the onset of autoacceleration. In applications of such resins as thin-film coatings, O_2_ diffusion can maintain a steady state of [O_2_] in the outer layer of a coating, as observed here in studies of a resin with Mn(II)(neodecanoate)_2_, where the rate of oxygen uptake from the headspace matches the rate of depletion due to the action of the catalyst. Hence, although inhibition of polymerization by O_2_ is not of relevance for systems such as those studied here under bulk conditions, the competition between diffusion of O_2_ from the atmosphere and consumption of O_2_ by the action of the catalyst on the initiator is likely to be relevant in determining the properties of such resins when used as coatings. This latter aspect is the focus of ongoing studies in our group.

## Experimental Section

Bisphenol-A-based bismethacrylate was provided by AkzoNobel, Sassenheim, for which the synthesis and characterization have been reported earlier.^24^ Styrene (≥99.0*%*), Co(II)(2-ethylhexanoate)_2_ solution (65 wt %), and cumene hydroperoxide (80%) were obtained from SigmaAldrich. The Al_2_O_3_ 90 active 70–230 mesh was obtained from Merck. All monomers were filtered over Al_2_O_3_ before use to remove stabilizers. Mn(II)(neodecanoate)_2_ (8 wt % on a metal basis) in mineral spirits and Fe(II)-bispidine (BorchiOXY-Coat 1410) were provided by Borchers. Resins were prepared by mixing BADGE-MA (bisphenol A-based diglycidyl ether dimethacrylate) and styrene. BADGE-MA was warmed in an oven at 80 °C for ca. 30 min. 10 g of warm BADGE-MA was poured into a disposable 20 mL glass vial with a screw cap, to which 3.5 g (i.e., 0.35 g/g BADGE-MA resin) of styrene was added, and a vortex mixer (Scientific Industries, Vortex Genie 2) or a SpeedMixer (FlackTek, DAC 330-100 SE) was used to homogenize the mixture. The mixture was allowed to cool to room temperature before use.

### Headspace Raman Spectroscopy

The composition of the gases in the closed headspace of quartz cuvette or glass vials was determined by Raman spectroscopy at λ_785_ as described earlier using the Raman bands of O_2_ and N_2_ at 1550 and 2320 cm^–1^.^25,26^ The concentration of dissolved oxygen in the alkenes used in the present study and in particular in the model mixture of styrene/methyl methacrylate/methoxy-2-propanol (1:1:1 by volume) and in BADGE-MA/styrene was estimated using calculated Henry’s law constant using the COSMO-RS method^45,46^ as implemented in AMS2024.^47^ The default settings were used to generate the .coskf files, BP86/TZP + ZORA, and “good” numerical quality. From the Raman measurement, oxygen partial pressure can be measured, and thus, the dissolved oxygen concentration can be calculated using
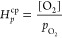


### Emission Spectroscopy and Phosphorescence Decay Lifetimes

A stock solution of 2 mg/mL [Ru(ph_2_phen)_3_](PF_6_)_2_ was prepared in acetonitrile for addition to the model mixture and the resin. Solutions of catalysts in mixtures of styrene/methyl methacrylate/methoxy-2-propanol (1:1:1 by volume) were prepared, and 1 mL (1.8 mL to avoid a headspace in the vial) was transferred into a 2 mL GC vial. 5 μL of the stock solution of [Ru(ph_2_phen)_3_](PF_6_)_2_ was added to the sample. BADGE-MA/styrene resin was prepared as described above, and the same concentration of [Ru(ph_2_phen)_3_](PF_6_)_2_, as used in the mixture of styrene/MMA/MeOPrOH, was added to the resin in a stock solution in acetonitrile. The vial was sealed with a GC-vial cap with a PTFE septum.

Cumene hydroperoxide (17 μL/ml) was added through the septum using a 50 μL Hamilton microliter syringe. Emission decay lifetimes were recorded using an FS-5 spectrofluorimeter by MCS (multichannel scaling) with a 450 nm (EPL-450) pulsed laser diode (Edinburgh Instruments).

### NIR Emission Spectroscopy

NIR emission spectra were recorded with a 355 nm CW laser (Cobolt lasers, 2 mW at sample) or 450 nm (45 mW, PowerTechnology) directed into the optical path of the spectrometer with a 45 ° long-pass dichroic beam splitter and focused onto the sample with a 25 mm diameter (*f* = 35 mm) planoconvex lens. Emission was collected by the same lens, passed through the dichroic beam splitter and a long-pass filter (1064 nm, Semrock) to remove visible light, and focused with a 25 mm diameter (*f* = 40 mm) planoconvex lens into a Shamrock 193i spectrograph equipped with an idus-InGaAs diode array (Andor Technology) with a 600 l/mm grating blazed at 860 nm. Spectra were recorded using Andor Solis and processed using Spectragryph 1.2.17.

#### Relation between the τ_obs_ of [Ru(ph_2_phen)_3_](PF_6_)_2_ and the Intensity of Emission from ^1^O_2_

The emission lifetime of [Ru(ph_2_phen)_3_](PF_6_)_2_ under air-equilibrated conditions in styrene/MMA/MeOPrOH is 315 ns, as expected considering that although the concentration of dissolved O_2_ (ca. 3.6 mM)^25,36,48^ is similar to CH_3_CN (τ = 169 ns), it is more viscous and hence molecular diffusivity is reduced. However, purging with argon gas increases the emission lifetime to 6.1 μs in CH_3_CN, which is consistent with values reported in the literature (namely, 6.3 μs in acetonitrile at 25 °C, degassed by four freeze–pump thaw cycles),^48^ and the lifetime only increased to 4.86 μs in the model mixture due to excited state deactivation by solvent, O–H oscillators.^49^ As expected, emission from ^1^O_2_ was not observed after argon purging either in CH_3_CN or the model solvent mixture. The ratio of Raman bands of O_2_ to N_2_ in the Raman spectrum recorded in the headspace above the model alkene solvent mixture was used, together with the Henry constant, to estimate the concentration of dissolved O_2_. As expected, a linear correlation between the concentration of dissolved O_2_ and the emission decay rate was obtained.

The limit of detection for emission from ^1^O_2_ was determined by increasing the concentration of ^3^O_2_ incrementally and determining both the emission lifetime of [Ru(ph_2_phen)_3_](PF_6_)_2_ and the intensity of the NIR emission from ^1^O_2_ (Figure S13); emission from ^1^O_2_ was above the limit of detection in samples where the emission lifetime of [Ru(ph_2_phen)_3_](PF_6_)_2_ was between 2.7 and 2.9 μs, and even at 3.9 μs, a distinct emission at 1268 nm was observable under the conditions employed here (Figure S8), which corresponds to less than 10% of the original [O_2_].

## Supporting Information Available

Additional discussion of polymerization kinetics, time-dependent UV/vis absorption, emission, emission decay data, alkene, and oxidant conversion over time, and calibration curves (PDF)
