# Supplementary material for: Dependence of O2 Depletion on Transition Metal Catalyst in Radical Polymerization of Cross-Linking Alkene Resins
Source: Inorg Chem. 2025 Apr 5;64(15):7716–25. doi: 10.1021/acs.inorgchem.5c00760 (PMC12015813; doi:10.1021/acs.inorgchem.5c00760)
Supplement: Supplementary file 1 — ic5c00760_si_001.pdf [file ic5c00760_si_001.pdf]

Supporting information

Dependence of O_2 depletion on transition metal catalyst in radical polymerization of crosslinking alkene resins

Hugo den Besten,[a] Yanrong Zhang,[a] Linda E. Eijsink,[a] Andy S. Sardjan,[a] Anouk Volker,[a] and Wesley R. Browne[a,*]

[a]Molecular Inorganic Chemistry, Stratingh Institute for Chemistry, Faculty of Science and Engineering, University of Groningen, Nijenborgh 3, 9747 AG, Groningen, The Netherlands

email: w.r.browne@rug.nl

The primary reactions of interest in radical polymerisations

The role of the catalysts is to generate radicals ($\mathbf{R}^{\cdot}$) from the the initiator (**I**) which can then react with monomer (alkene) to form a new (propagating) radical (3a), which itself reacts again with an alkene monomer (3b). Two processes of interest in the current study are (i) autoacceleration and (ii) the impact of oxygen on the polymerization. (i) **Autoacceleration.** The generation of radicals ($\mathbf{R}^{\cdot}$) from the the initiator (**I**) produces growing polymer chains, however, at low viscosity the radicals have enough time to meet other radicals and react, resulting in termination of polymerization. Eventually the solution viscosity increases sufficiently to limit this termination process and the number of growing polymer chains increases, resulting in a rapid increase in monomer (alkene) conversion to polymer. This increase in rate is referred to as the Trommsdorff or autoacceleration phase. The delay in the onset of this phase is essential in applying such resins as coatings as it allows time between mixing of resin with initiator and application of the coating).

(ii) **Impact of $\mathbf{O_2}$ on polymerization.** The presence of molecular oxygen also leads to inhibition of polymerization. The rate of addition of alkene monomer (**M**) to a growing chain, k_2 - eq. 3b, is ca. 10^2 l mol^{-1} s^{-1}, is orders of magnitude lower than that of the reaction of chain end radicals with O_2, eq. 5 (10^8 l mol^{-1} s^{-1}). If O_2 is present then relatively stable peroxy radicals can form. Eq. 6 describes the rate determining step in the chain propagation with k_4 = ca. 10^{-1} l mol^{-1} s^{-1}. The newly formed alkyl radical (Equation 6) can still react with O_2 present since $k_3 \approx k_5$, eq. 7. The inhibition period observed in free radical polymerization lasts at least for as long as dissolved O_2 is present and a co-polymer between oxygen and monomer is formed. However it should be noted that certain chain termination steps can result in O_2 release also, eq. 8.

In summary, inhibition by O_2 is due to the retardation of polymerization, $k_4 \ll k_2$, while the termination step, *e.g.*, eq. 8, is of the same order of magnitude as termination by combination of alkyl radicals. Hence, the ratio of chain termination to chain propagation

in the presence of O_2 ($10^4/10^{-1} = 10^5$) is much higher compared to propagation when the concentrations of radicals and O_2 are similar ($10^4/10^2 = 10^2$), resulting in short chains, low concentration of radicals and inhibition of polymerization

$$\mathrm{I} \xrightarrow{\mathrm{ki}} 2\,\mathrm{R}^{\bullet} \tag{2}$$

$$\mathrm{R}^{\bullet} \xrightarrow{\mathrm{k_1}} \mathrm{RM}^{\bullet} \tag{3a}$$

$$\mathrm{RM}^{\bullet} \xrightarrow{\mathrm{k_2}} \mathrm{RMM}^{\bullet} \tag{3b}$$

$$\mathrm{RM}^{\bullet} + \mathrm{RM}^{\bullet} \xrightarrow{\mathrm{k}_t} \mathrm{RMMR} \tag{4}$$

$$\mathrm{R}^{\bullet} + \mathrm{O_2} \xrightarrow{\mathrm{k_3}} \mathrm{RO_2}^{\bullet} \tag{5}$$

$$\mathrm{RO_2}^{\bullet} + \mathrm{M} \xrightarrow{\mathrm{k_4}} \mathrm{RO_2M}^{\bullet} \tag{6}$$

$$\mathrm{RO_2M}^{\bullet} + \mathrm{O_2} \xrightarrow{\mathrm{k_5}} \mathrm{RO_2MO_2}^{\bullet} \tag{7}$$

$$\mathrm{RO_2(MO_2)}_n{}^{\bullet} + \mathrm{RO_2(MO_2)}_m{}^{\bullet} \xrightarrow{\mathrm{k_6}} \mathrm{(Co-)polymer} + \mathrm{O_2} \tag{8}$$

Additional spectroscopy and kinetic data

Cumene hydroperoxide decomposition rates

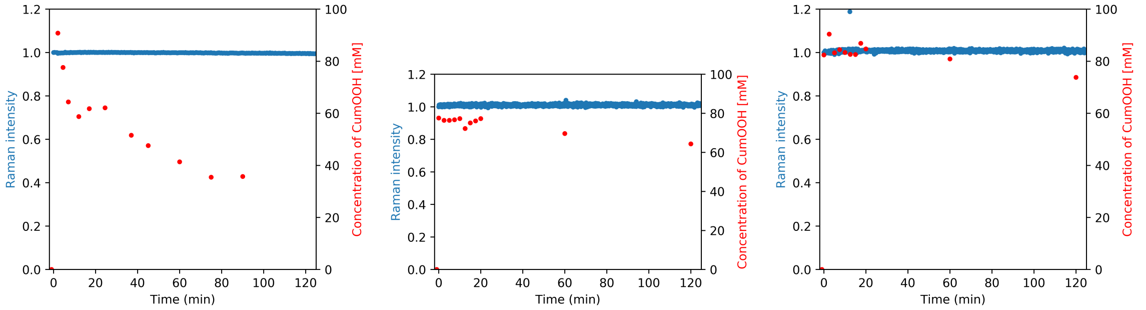

Figure S1: Alkene conversion monitored by in situ Raman spectroscopy (λ_{exc} 785 nm, integrated area of Raman band at 1614-1675 cm^{-1}, normalized to initial area) concurrent with concentration of cumene hydroperoxide (CumOOH, initial: 92 mMolal) over time determined by iodine liberation in styrene/MMA. Decomposition catalyzed by Co(II)(2-ethylhexanoate)$_2$ (top), Fe(II)-bispidine (middle) and Mn(II)(neodecanoate)$_2$ (bottom).

O_2 solubility

Table 1: Henry's constant and calculated concentrations of O_2 in the solvents and mixtures discussed in the text

Solvent system	Henry's Constant (mol/(L atm))	$[O_2](P_{O_2} = 1$ atm) (mM)	$[O_2](P_{O_2} = 0.21$ atm) (mM)
MMA	1.87×10^{-2}	18.67	3.92
Styrene	1.88×10^{-2}	18.79	3.95
1-Methyl-2-propanol	1.50×10^{-2}	15.04	3.16
MMA:styrene:MeOPrOH[a]	1.71×10^{-2}	17.14	3.60
MMA:t-butylstyrene:MeOPrOH[b]	1.63×10^{-2}	16.28	3.42
BADGE-MA	5.32×10^{-3}	5.32	1.12
BADGE-MA:styrene[c]	9.13×10^{-3}	9.13	1.917

[a] 1:1 by volume

[b] 1:1:1 by volume

[c] 1:1 by double bonds

Emission decay and alkene conversion over time.

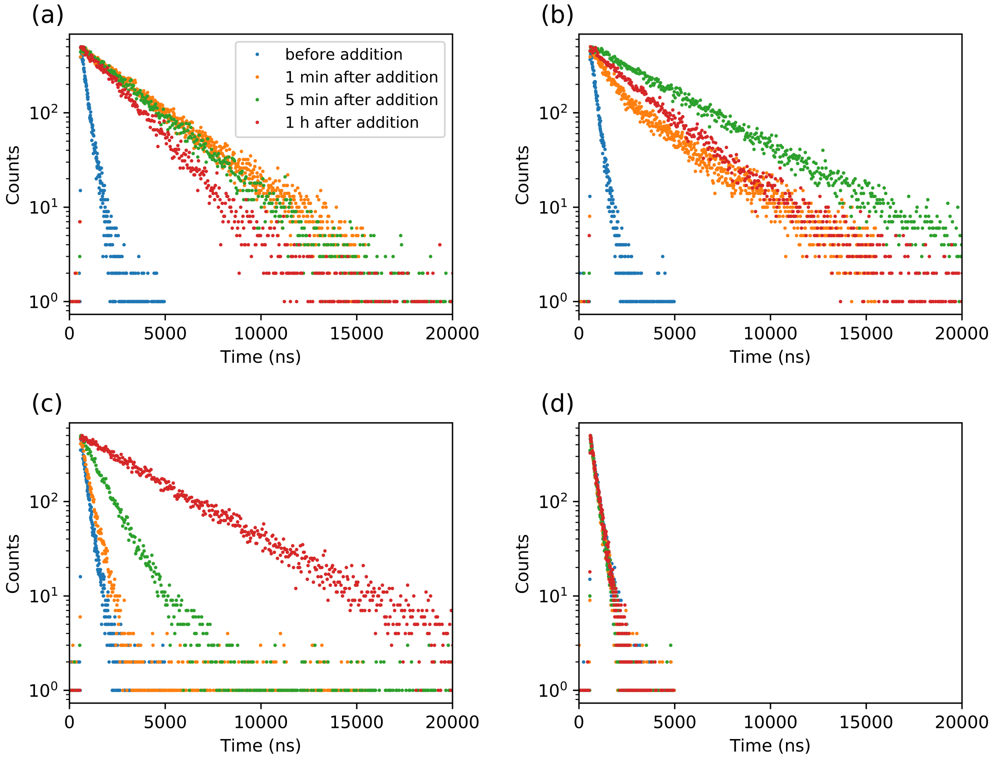

Figure S2: Emission decay of $[Ru(ph_2phen)_3](PF_6)_2$ (λ_{exc} 450 nm) in styrene/MMA/methoxy-propanol containing a) $Co(II)(2\text{-ethylhexanoate})_2$ (2 mM), b) Fe(II)-bispidine (0.2 mM), c) $Mn(II)(neodecanoate)_2$ (2 mM), and d) without catalyst, before and 2 min, 5 min and 1 h after addition of cumene hydroperoxide (92 mM).

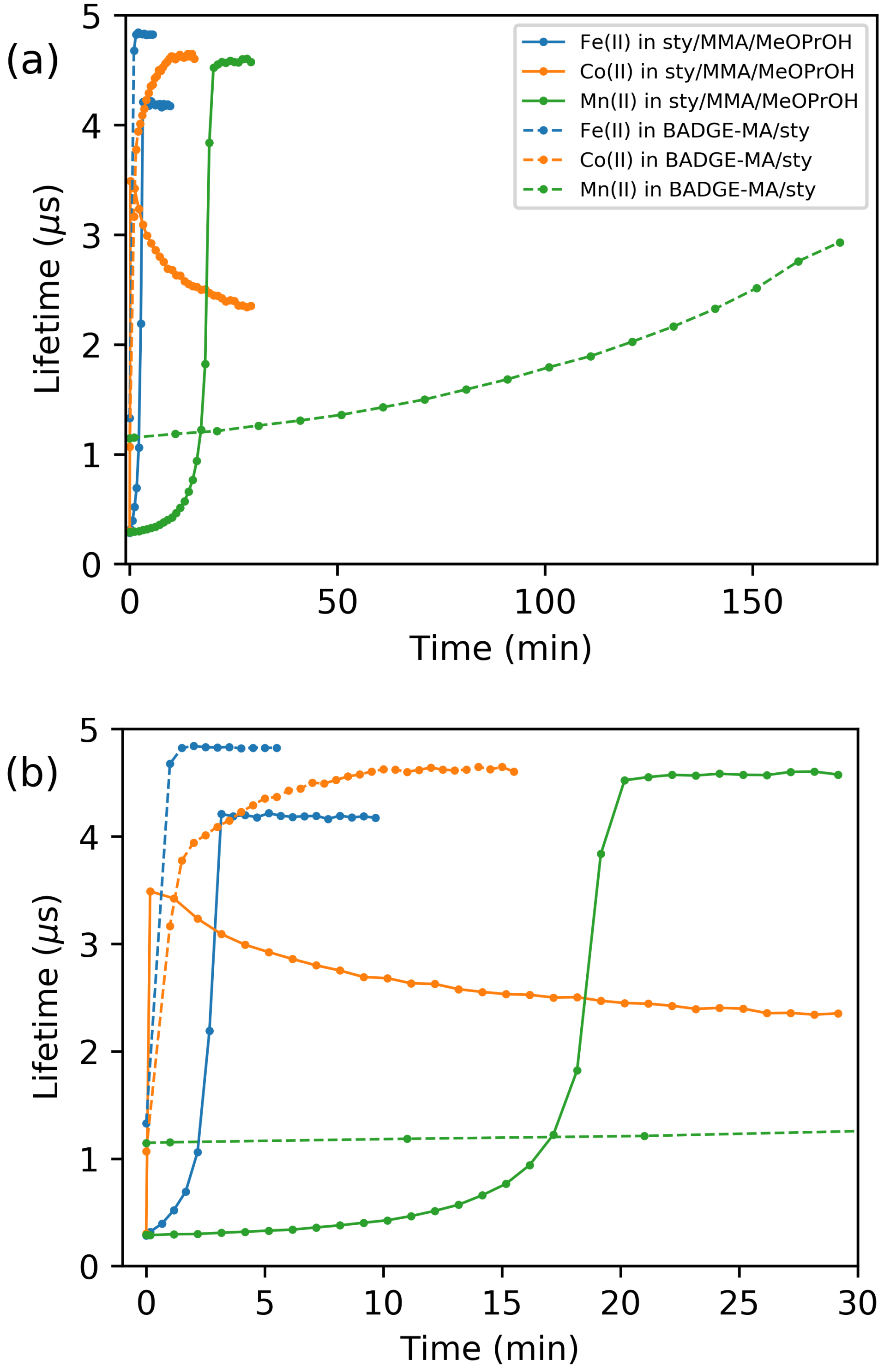

Figure S3: (a) Emission lifetime (τ_{obs}) of $[Ru(ph_2phen)_3](PF_6)_2$ with Fe(II)-bispidine (blue), Co(II)(2-ethylhexanoate)$_2$ (red), or Mn(II)(neodecanoate)$_2$ (green, right shows over 3 h range in resin), in styrene/MMA/methoxy-propanol (solid lines) and in BADGE-MA/St resin (dashed lines) after addition of cumene hydroperoxide (92 mM). (b) expansion showing first 30 min

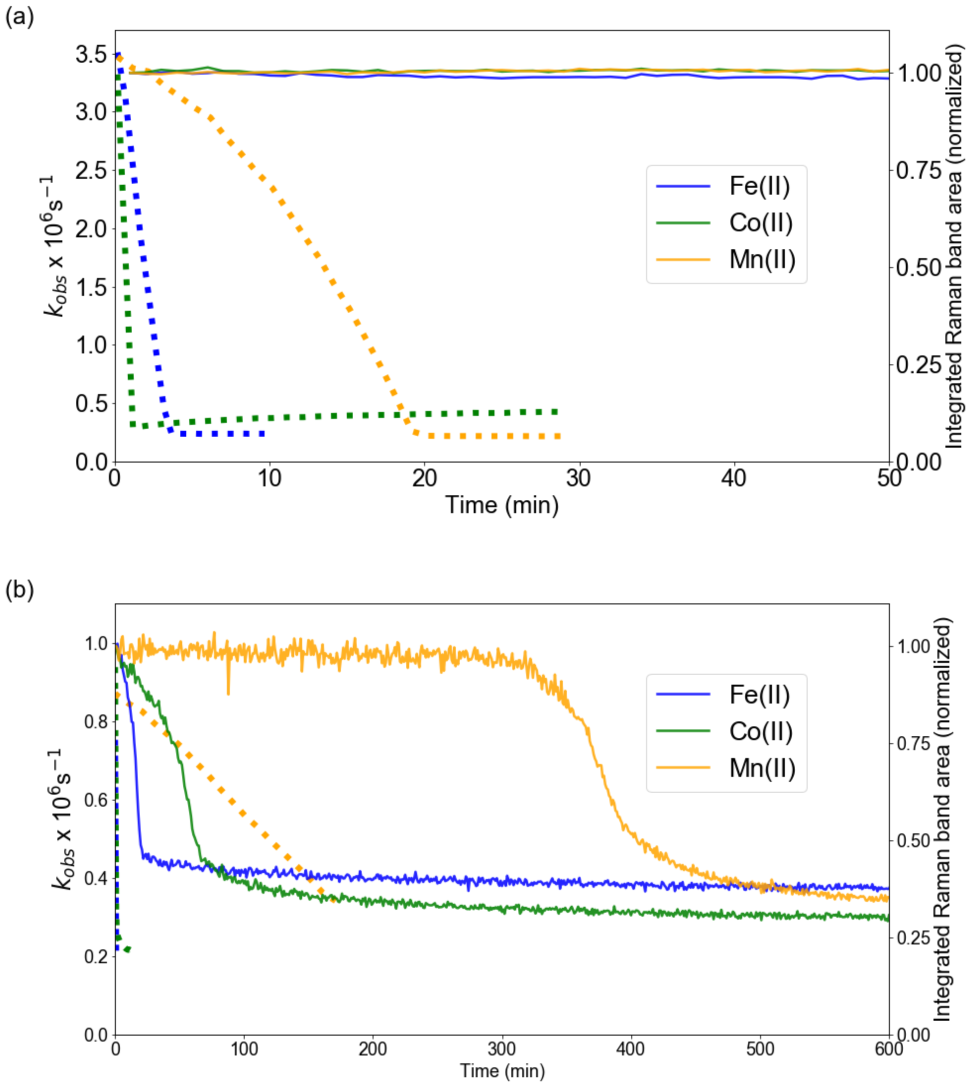

Figure S4: k_{obs} (squares) and and area of the $\nu_{C=C,str}$ (normalised to initial area) Raman band at 1630-1637 cm^{-1} over time after addition of cumene hydroperoxide (92 mM) with Fe(II)bispidine, Co(II)(2-ethylhexanoate)$_2$, or Mn(II)(neodecanoate)$_2$ (a) in styrene/MMA/MeOPrOH and (b) in BADGE-MA/styrene resin.

UV/Vis absorption spectroscopy

UV/vis absorption spectra of catalysts in styrene/MMA/methoxy-propanol following addition of cumene hydroperoxide

The change in UV/vis absorption spectra after addition of cumene hydroperoxide in styrene/MMA/methoxy-propanol containing each of the catalysts show that, in the case of Co(II)(2-ethylhexanoate)$_2$, the absorbance increases at 450 nm over time with the major change in the first few minutes. For Mn(II)(neodecanoate)$_2$, the increase was much less pronounced, which for Fe(II)-bispidine the spectrum was essentially unchanged. Furthermore for all three complexes the absorbance at 1268 nm (wavelength of emission from $^1O_2$) is negligible and hence the major contribution to changes in emission intensity (both for $[Ru(ph_2phen)_3]^{2+}$ and $^1O_2$) is the primary inner filter effect. It is of note that the inner filter effect does not affect emission lifetime. Further than optical considerations, the changes in the spectrum of Co(II)(2-ethylhexanoate)$_2$ indicate an increase in oxidation state (Co(II) to Co(III)) during the early phase of the reaction but for Fe(II)-bispidine, in contrast, the complex remains in the Fe(II) state. The later observation suggests that despite the addition of cumene hydroperoxide, the solvent mixture has a low redox potential.

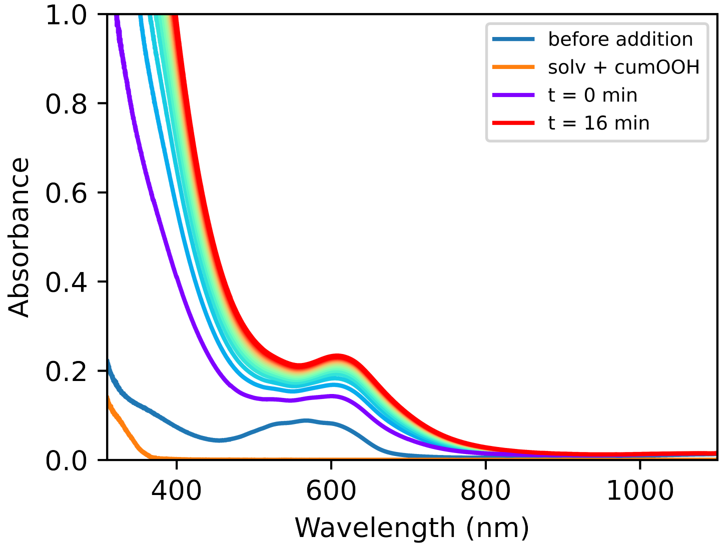

Figure S5: UV/vis absorption spectra of Co(II)(2-ethylhexanoate)$_2$ (1.9 mM) in styrene/MMA/methoxy-propanol before and after addition of cumene hydroperoxide (92 mM). Spectra recorded at 1 min intervals. Time indicates time after addition of initator

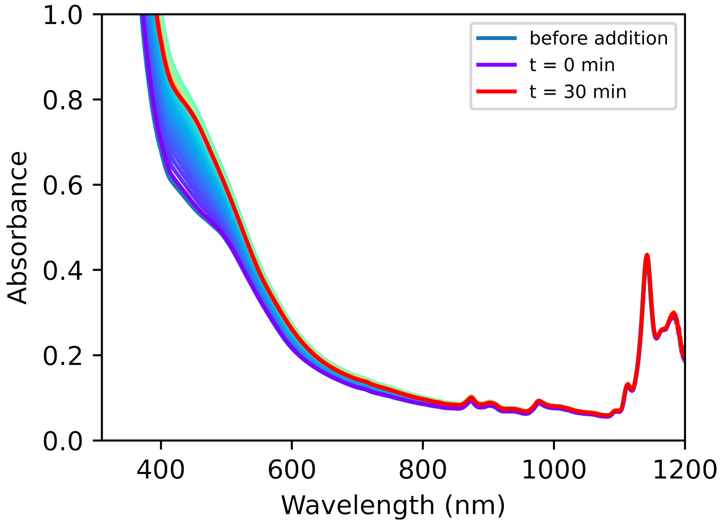

Figure S6: UV/vis absorption spectra of Mn(II)(neodecanoate)$_2$ (2.0 mM) in styrene/MMA/methoxy-propanol before and after addition of cumene hydroperoxide (92 mM). Spectra recorded at 1 min intervals.

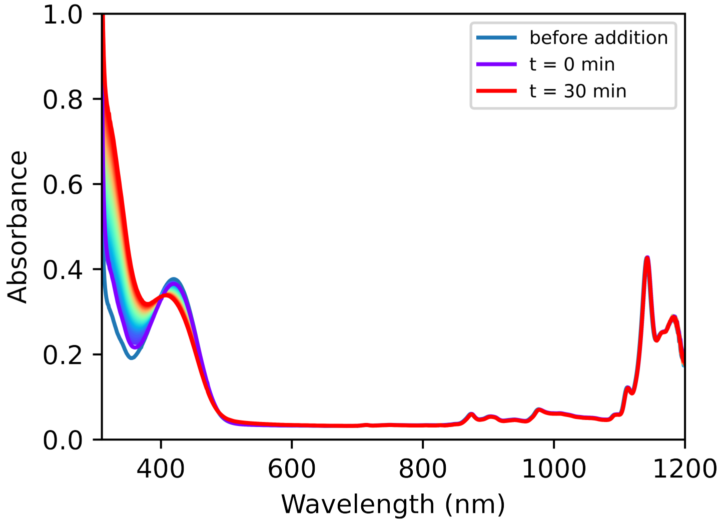

Figure S7: UV/vis absorption spectra of Fe(II)-bispidine (0.2 mM) in styrene/MMA/methoxy-propanol before and after addition of cumene hydroperoxide (92 mM). Spectra recorded at 1 min intervals. Spectra are recorded with air as reference. Narrow bands in the near infra-red are vibrational overtones and combination bands of the solvent.

NIR emission spectroscopy

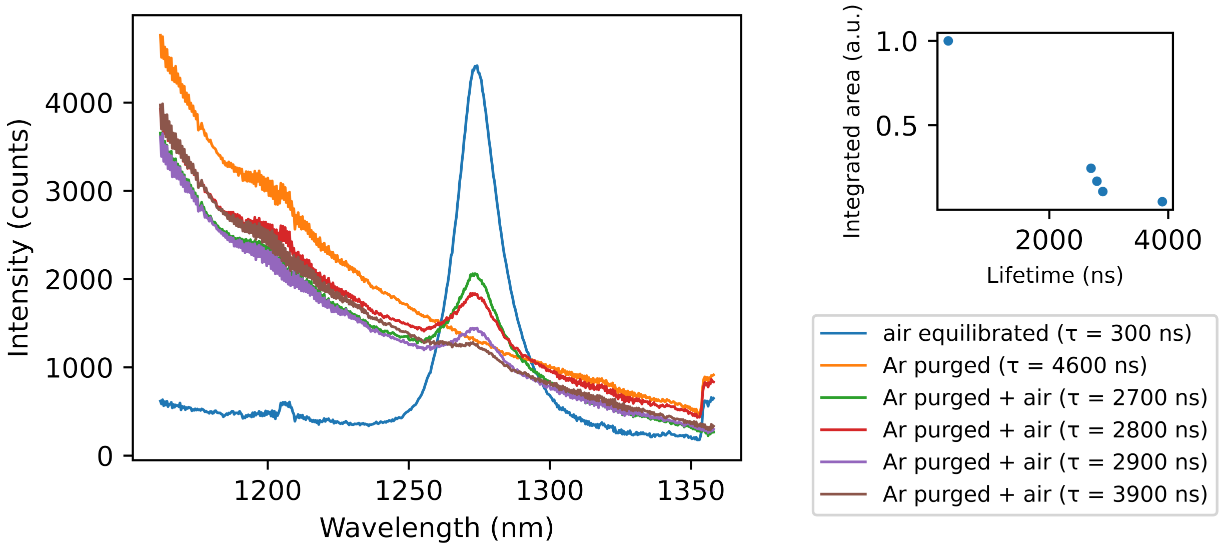

Figure S8: Emission from $^1O_2$ generated by sensitization with $[Ru(ph_2phen)_3](PF_6)_2$ in styrene/MMA/methoxy-propanol, before and after purging with argon and then air. Corresponding lifetimes indicated for each measurement, (λ_{exc} 450 nm). Integration of each band plotted vs. emission lifetime (top right).

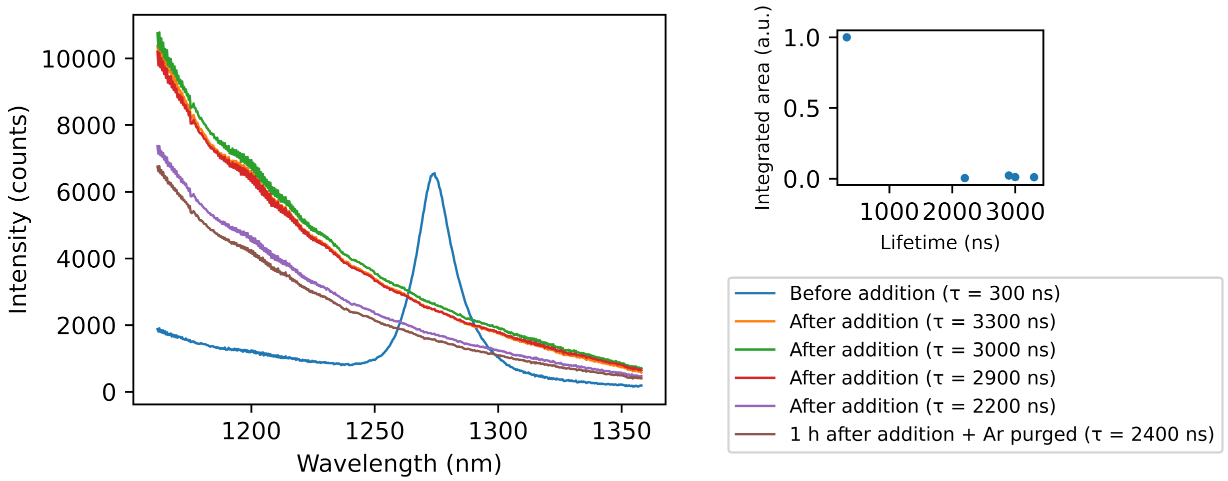

Figure S9: Emission from $^1O_2$ and the tail of emission of $[Ru(ph_2phen)_3](PF_6)_2$ in styrene/MMA/methoxy-propanol containing Co(II)(2-ethylhexanoate)$_2$ (1.88 mM) before and after addition of cumene hydroperoxide (92 mM), (λ_{exc} 450 nm). Corresponding lifetimes indicated for each measurement. Emission (integrated area) at 1268 nm vs. lifetime (top right).

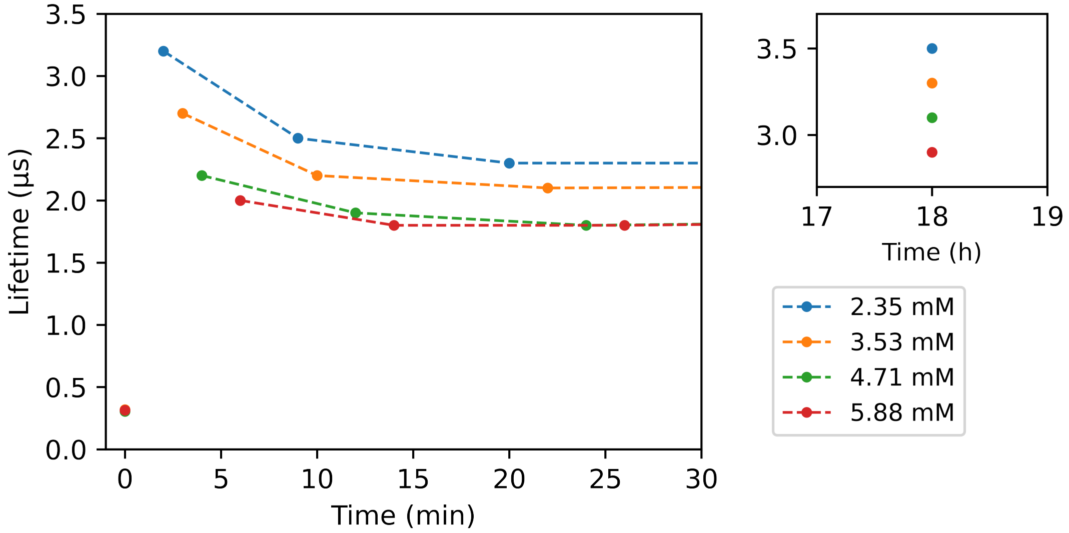

Figure S10: Emission lifetime of $[Ru(ph_2phen)_3](PF_6)_2$ in styrene/MMA/methoxy-propanol before and after addition of cumene hydroperoxide, with various concentrations of cobalt catalyst. Note that lifetimes for each catalyst concentration were obtained sequentially and hence at different times after addition of initiator. Dotted lines were added to guide the eye. Emission lifetimes after 18 h shown top right.

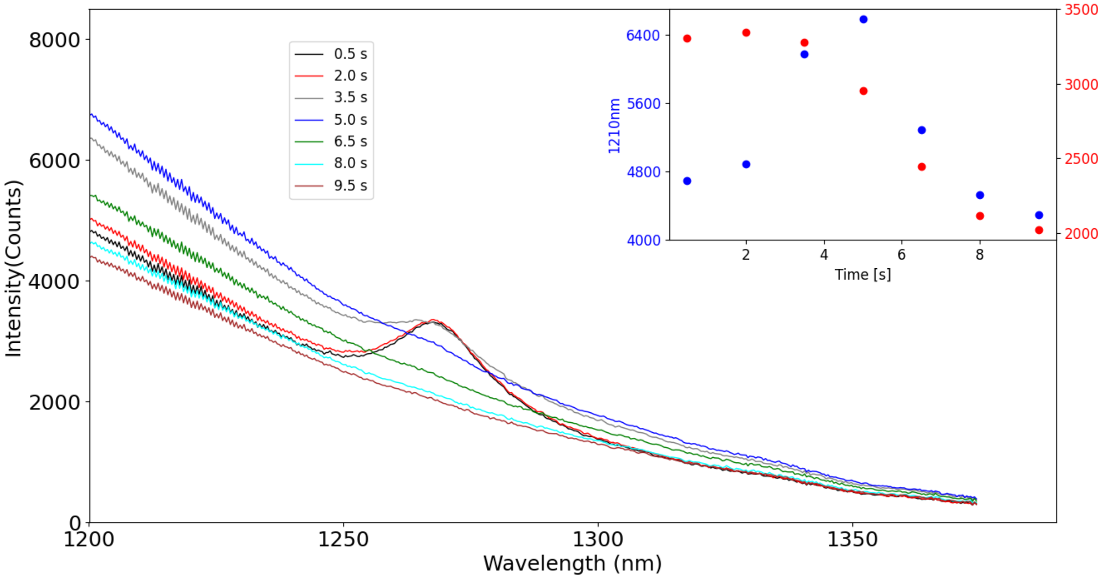

Figure S11: NIR emission from a resin containing $[Ru(ph_2phen)_3](PF_6)_2$ over time (spectra recorded each 0.5 s). Initially both the broad emission from $[Ru(ph_2phen)_3](PF_6)_2$ and narrow emission of $^1O_2$ at 1268 nm is observed. The emission of $[Ru(ph_2phen)_3](PF_6)_2$ increases rapidly (inset blue) concomitant with a disappearance of the emission of $^1O_2$ (inset red) and thereafter the emission of $[Ru(ph_2phen)_3](PF_6)_2$ begins to decrease also indicating photobleaching. λ_{exc} 450 nm, 20 mW at sample.

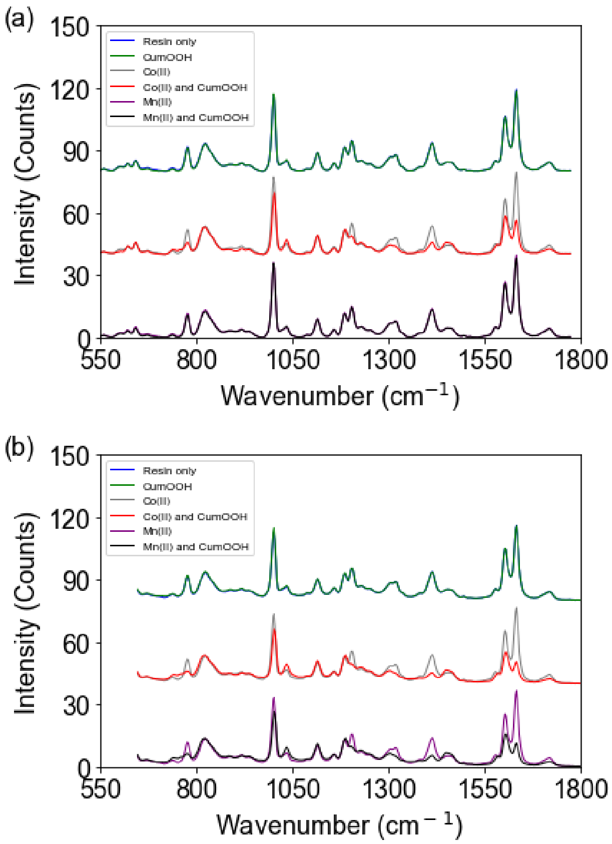

Figure S12: Raman spectra at λ_{exc} 785 nm of BADGE-MA/styrene resin used in figure 11 (a) 2h and (b) 24 h after addition of cumene hydroperoxide, Co(II)(2-ethylhexanoate)$_2$, Mn(II)(neodecanoate)$_2$, Co(II)(2-ethylhexanoate)$_2$ with cumene hydroperoxide, Mn(II)(neodecanoate)$_2$ with cumene hydroperoxide, and without catalyst or cumene hydroperoxide.

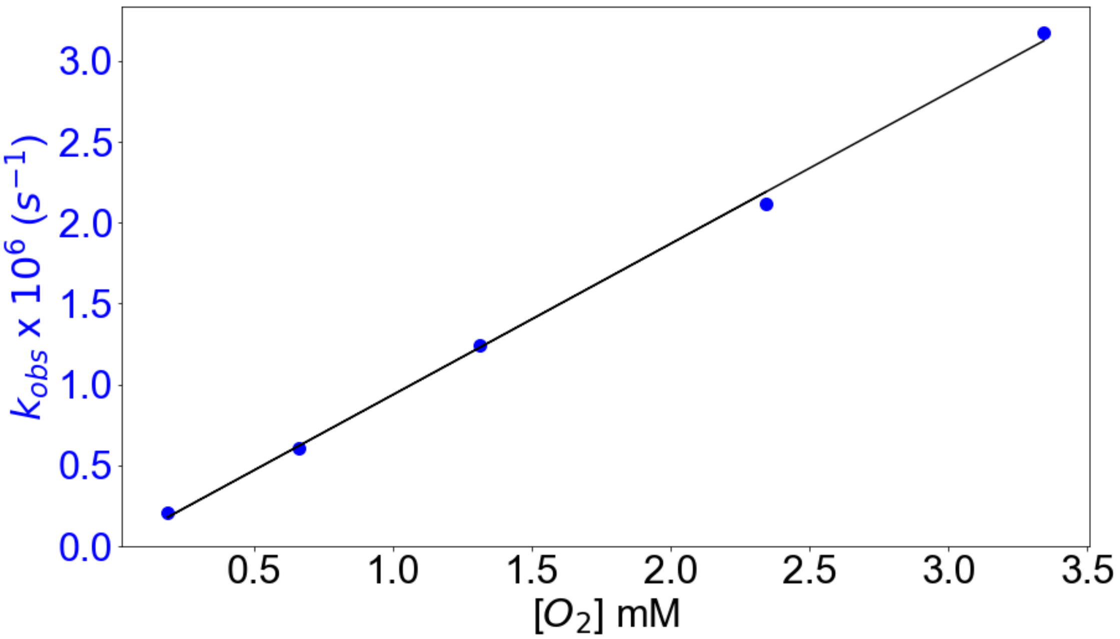

Figure S13: Dependence of the decay rate of the emission from $[Ru(ph_2phen)_3]^{2+}$ on [O_2], in styrene/methyl methacrylate/methoxy-2-propanol (1:1:1 by volume). The [O_2] was determined by determination of partial pressure of O_2 in the equilibrated headspace by Raman spectroscopy. (black line) Linear regression : $k_o bs(s^{-1})$= 9.34×10^8 (M^{-1} s^{-1}) $\times$ [O_2] (mM) + 1682 (s^{-1})

Calibration curve for iodine liberation by reaction with cumene hydroperoxide

230 mg of cumene hydroperoxide was added to a 1:1 (v/v) mixture of styrene and methyl methacrylate (MMA), which had both passed over AlOx to remove inhibitor, yielding a final volume of 10.0 mL (with 120 mM cumene hydroperoxide). A set of 12 dilutions were prepared, ranging from undiluted styrene/MMA with 120 mM cumene hydroperoxide to styrene/MMA without cumene hydroperoxide. The cumene hydroperoxide concentration was determined using the method described ealier (Eijsink et al. Cat. Sci. Tech. 2024, 14, 2423-2433). The resulting calibration curve is shown in Figure S14.

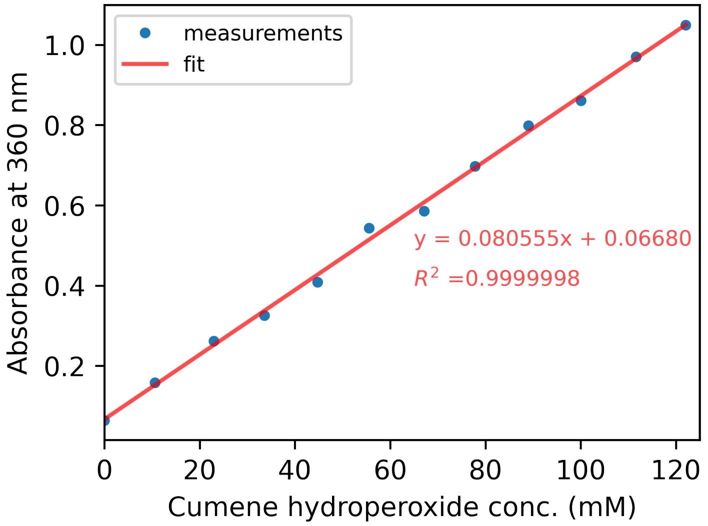

Figure S14: Calibration curve for determination of cumene hydroperoxide by iodine liberation.
